# Effect of Interspecific Yeast Hybrids for Secondary In-Bottle Alcoholic Fermentation of English Sparkling Wines

**DOI:** 10.3390/foods12101995

**Published:** 2023-05-15

**Authors:** Matteo Marangon, Poppy Seeley, Erica Barocci, Tony Milanowski, Christine Mayr Marangon, Arianna Ricci, Jennifer Bellon, Giuseppina P. Parpinello

**Affiliations:** 1 Department of Agronomy, Food, Natural Resources Animals and Environment (DAFNAE), University of Padua, Viale dell’Università, 16, 35020 Legnaro, Italy; 2 Interdepartmental Centre for Research in Viticulture and Enology (CIRVE), University of Padova, via XXVIII Aprile 14, 31015 Conegliano, Italy; 3 Wine Division, Plumpton College, Ditchling Road, Lewes BN7 3AE, UK; 4 Department of Agricultural and Food Sciences (DISTAL), University of Bologna, Piazza Goidanich, 60, 47523 Cesena, Italy; 5 The Australian Wine Research Institute, Glen Osmond, P.O. Box 197, Adelaide, SA 5064, Australia

**Keywords:** sparkling wine, interspecific yeast hybrids, foam, phenolics, macromolecules, sensory

## Abstract

In sparkling winemaking, only a few yeast strains are regularly used for the secondary in-bottle alcoholic fermentation (SiBAF). Recently, advances in yeast development programs have yielded new breeds of interspecific wine yeast hybrids that ferment efficiently while producing novel flavors and aromas. In this work, the chemical and sensorial impacts of the use of interspecific yeast hybrids for SiBAF were studied using three commercial English base wines prepared for SiBAF using two commercial and four novel interspecific hybrids. After 12 months of lees aging, the chemical and macromolecular composition, phenolic profile, foaming, viscosity and sensory properties of the resulting 13 wines were assessed. Chemically, the yeast strains did not result in significant differences in the main wine parameters, while some differences in their macromolecular contents and sensory characteristics were noticeable. The foamability was mostly unaffected by the strain used; however, some effect on the foam stability was noticeable, likely due to the differences in polysaccharides released into the wines by the yeast strains. The wines exhibited different sensory characteristics in terms of aroma and bouquet, balance, finish, overall liking and preference, but these were mostly attributable to the differences in the base wines rather than the strain used for SiBAF. Novel interspecific yeast hybrids can be used for the elaboration of sparkling wines, as they provided wines with chemical characteristics, flavor and aroma attributes similar to those of commonly used commercial *Saccharomyces cerevisiae* strains.

## 1. Introduction

The International Organisation of Vine and Wine (OIV) categorizes sparkling wines into the special wines section as products that, upon uncorking, produce an effervescence that results in foam formation constituted by bubbles of carbon dioxide derived from a secondary alcoholic fermentation process that can take place inside a bottle (bottle-fermented) or in a closed tank (tank-fermented) [1]. The popularity of this type of wines has been steadily increasing in the past two decades to reach 20 mhl in 2018 [2].

In sparkling winemaking, a variety of yeasts can be used to conduct the primary alcoholic fermentation that leads to the elaboration of the base wine required for conducting a second alcoholic in-bottle fermentation (SiBAF). However, the second fermentation is challenging for the yeasts due to the characteristics of base wines: high alcohol content (around 11%), low sugar (20–24 g/L), with both low pH (generally below 3.20) and temperature (range 12–16 °C), in the presence of some SO_2_ and with increasing pressure due to the CO_2_ formed during the fermentation [3]. This means that only a few selected *Saccharomyces cerevisiae* yeast strains are suitable to carry out this task and are therefore regularly used for the secondary in-bottle alcoholic fermentation [4]. However, it is widely known that different yeasts can impart different characteristics to wine [5]; hence, having more strains available for sparkling wine production would allow enologists to diversify their products, thus increasing the offer for consumers [6]. Indeed, the interest for this wine typology has increased in recent years (e.g., Prosecco “phenomenon”), with several studies reporting on the differences induced by a secondary fermentation with alternative yeast strains in compounds as amino acids and ammonia, volatile aroma compounds (VOCs), glycerol, organic acids, proteins and polysaccharides [4,6,7,8,9].

An interesting venue to increase the availability of yeast strains for sparkling wine production comes from recent advances in yeast development programs that resulted in new breeds of interspecific wine yeast hybrids that ferment efficiently while producing novel flavors and aromas [10,11]. *Saccharomyces cerevisiae* is the typical yeast species chosen for the inoculation of grape juice due to specialist strains having impressive tolerances against the high sugar and ethanol levels that occur during wine production. While some other members of the *Saccharomyces* species have been isolated from wine fermentations, their winemaking abilities are generally considered inferior to their *cerevisiae* sibling. The *Saccharomyces* species are a genetically diverse genus but share a common mating system. Natural, interspecific mating between a robust wine yeast strain and other members of the *Saccharomyces* clade has allowed researchers to build a series of wine yeast strains with the same *Saccharomyces cerevisiae* parent but with genomic contributions from a different *Saccharomyces* species. Combining these divergent genomes into one organism has led to the generation of new yeast strains with robust fermentation traits and the added flavor-active metabolites of the non-*cerevisiae* genome component. The *Saccharomyces cerevisiae* parent strain of the interspecific hybrids used in this study is a member of the PdM clade of highly successful wine yeast strains, of which the champagne yeast IOC 18-2007 is also a member [12]. This work aims at enlarging the available yeast strains for sparkling wine production; with this aim, secondary in-bottle alcoholic fermentation with conventional and novel interspecific hybrids yeast strains was carried out, and the chemical and macromolecular compositions, phenolic profiles, foaming and viscosity properties were assessed after a 1-year period of aging on lees from 13 wines.

## 2. Materials and Methods

### 2.1. Materials and Experimental Design

Three base wines were kindly donated by wineries from the southeast of England. All the wines were produced commercially using different combinations of traditional grape varieties (Chardonnay, Pinot noir and Pinot meunier). Base wine A was a blend of Chardonnay (41%), Pinot meunier (41%) and Pinot noir (18%); base wine B was mostly made of Chardonnay (98%) with some Pinot noir (2%); base wine C, the only rosé wine of the three, was a blend of Pinot noir (97%) and Pinot meunier (3%).

The primary fermentation for wine A was carried out using the yeast IOC 18-2007 (Institut Œnologique de Champagne, Mardeuil, France), while both wines B and C were fermented with a blend of three yeasts for the primary fermentation: Shia Varioferm (45%) (Eaton, Nettersheim, Germany), IOC Divine (35%) and IOC 18-2007 (20%) (Institut Œnologique de Champagne, Mardeuil, France). The base wines were all cold-stabilized and earth-filtered (1 µm), and their free SO_2_ was adjusted to 15 mg/L.

For the secondary in-bottle alcoholic fermentation (SiBAF), the three base wines were prepared for tirage 8 months after harvest by adding 24 g/L of sucrose, 50 mg/L of phosphate titrés as a nitrogen supplement (IOC) and 6 mL/L of Clariant XL (IOC) as the clarifying agent and by using different yeast strains, which characteristics are shown in Table 1. A total of 50 bottles were prepared for each of the analyzed yeasts strains.

**Figure 1:**
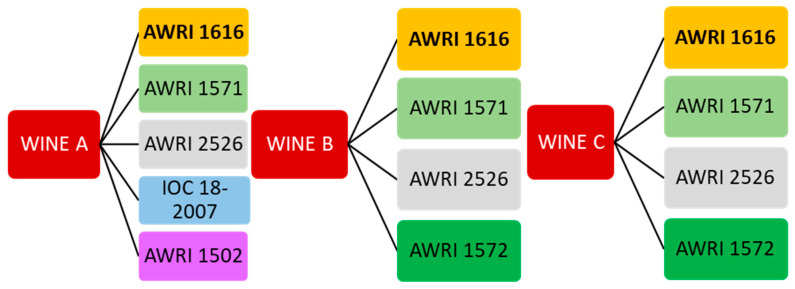
Schematic representation of the yeasts used for the secondary in-bottle alcoholic fermentation of the three base wines.

The experimental design is shown in Figure 1. The commercial strain AWRI 1616 was used in all wines as the reference yeast due to its large diffusion in the production of bottle-fermented sparkling wines. Given that the wines were commercially produced, the enologists involved in the experiments could select which and how many yeast strains to use during tirage. This resulted in two interspecific yeast hybrids (AWRI 1571 and AWRI 2526) being used in each of the three base wines, while three additional strains (IOC 18-2007, AWRI 1502 and AWRI 1572) were used in the selected wines following a decision with the enologists of the wineries providing the wines.

During SiBAF, the bottles were stored horizontally at 12–14 °C for 12 months of lees aging before being riddled and disgorged, as previously described [13]. Disgorged wines were added with 30 ppm of SO_2_ (as potassium metabisulphite, Enartis, San Martino, Italy) and immediately sealed with a crown cap.

### 2.2. Analytical Methods

The alcohol strength (% *v*/*v*) was measured according to the official OIV method [1]. The wine pH (units) and titratable acidity (g/L of tartaric acid equivalent) were determined on previously degassed wines at 25 °C by using an autotitrator (TitroLine easy, SI Analytics—Xilem brand, Weilheim, Germany [14]). The free and total SO_2_ (mg/L) were measured by using the aspiration method [14]. The wine color (AU) was assessed by using the tristimulus method CIELAB with a Hach Lange LICO 500 colorimeter (Keison Products, Chelmsford, UK) [15]. The glucose and fructose concentrations (g/L) were measured enzymatically using the commercial kit Randox via a RX Monaco analyzer (Randox, Crumlin, UK) [1].

### 2.3. Total Protein, Polysaccharides and Mannoprotein Quantification

The total protein content of the wines was determined using the potassium dodecyl sulfate (KDS) method [16]. The total polysaccharide content was determined colorimetrically by using an adaptation of the method from Segarra et al. [17], as reported in detail by Marassi et al. [18]. The wine mannoprotein content was assessed by using the Competitive Indirect Enzyme-Linked Lectin Sorbent Assay (CI-ELLSA), as previously described [19].

### 2.4. Total Phenolic Content

The total wine phenolic content was determined spectrophotometrically on degassed samples and diluted 1:11 with a model wine solution (0.5% *w/v* tartaric acid in 12% *v/v* ethanol adjusted to pH 3.4 with 5 M NaOH), and 1:51 with 1 M HCl as suggested by Mercurio et al. [20]. The obtained values were expressed in absorbance units (a.u.), as proposed by Iland et al. [14]. The phenolic parameters were calculated as follows:

- total phenolics (a.u.): ((Abs 280 nm * 51) − 4);
- total hydroxycinnamates (a.u.): ((Abs 320 nm * 11) − 1.4);
- total flavonoids (a.u.): [((Abs 280 nm * 51) − 4)) − ((0.66 * Abs 320 nm * 11) − 1.4)];
- brown pigments (a.u.): (Abs 420 nm * 11).

### 2.5. Determination of Phenolic Compounds by High-Performance Liquid Chromatography

The determination of phenolic compounds was conducted, as previously reported [21], using a Dionex DX-600 HPLC equipped with a DAD detector (Thermo Scientific, Waltham, MA, USA). All samples were degassed and sterile-filtered (0.22 μm) prior to injection. The separation of benzoic acids, hydroxycinnamic acids and flavonoids was performed by using an Inersustain C18 column (5 μm, 4.6 × 250 mm, CPS Analitica, Milano, Italy) as the stationary phase. The different compounds were eluted by applying a binary gradient (buffer A: 5% CH_3_COOH in 5% water, *v/v*; buffer B: 80% acetonitrile: 20% water, *v*/*v*). The injection volume was 10 μL. The identification and quantification of different phenolic compounds was performed by using different standard calibration curves with protocatechuic acid, vanillic acid, hydroxybenzoic acid, gallic acid, syringic acid, (+)-catechin, (−)-epicatechin, p-coumaric acid, caffeic acid, chlorogenic acid, ferulic acid, rutin and quercetin. All standards were sourced from Sigma-Aldrich (St. Louis, MO, USA). The compounds for which a standard was unavailable (caftaric, sinapic, coutaric and ferulic acids) were quantified as coumaric (CUE) or caffeic (CAE) acid equivalents.

### 2.6. Organic Acids by High-Performance Liquid Chromatography

The concentration of organic acids (citric, tartaric, malic, succinic and lactic) was determined as described previously [22] using an Aminex HPX-87H Bio-Rad 7.8 × 300 mm column (Bio-Rad, Hercules, CA, USA) fitted on a Dionex DX-600 HPLC equipped with a DAD detector (Thermo Scientific, Waltham, MA, USA).

### 2.7. Sodium Dodecyl Sulfate-Polyacrylamide Gel Electrophoresis (SDS-PAGE)

SDS-PAGE for the detection of proteins and glycoproteins was performed according to Laemmli [23] in a Mini-Protean III electrophoresis cell (Bio-Rad Laboratories, Hercules, CA, USA). Samples were prepared by precipitating proteins and glycoproteins from 400 µL of wine using four volumes of cold ethanol. Pellets were collected by centrifugation (13,000× *g*, 15 min, 4 °C) and washed with 1 mL of pure ethanol before being dissolved in 25 μL of Laemmli sample buffer (Bio-Rad) prepared with 5% (*v*/*v*) 2-mercaptoethanol as the reducing agent. The samples were then heated at 95 °C in a dry bath (H_2_O_3_–100C, Coyote Bioscience Co., Ltd., Beijing, China) for 5 min. Then, 10 μL of each sample were loaded on Mini-Protean TGX stain-free precast gels 8–16% (Bio-Rad Laboratories). The Precision Plus Protein Standards broad range (range 10–250 kDa, Bio-Rad Laboratories) was used. Proteins were stained by using the silver stain procedure [24] and periodic acid–Schiff (PAS) for glycoprotein quantification [25]. Images of the gels were acquired at 300 dpi resolution with a ChemiDoc™ XRS molecular imager (Bio-Rad Laboratories).

### 2.8. Foaming Properties

A modified version of the Mosalux method was used to analyze the foaming properties of the wines [13,26,27]. The wine samples were firstly degassed in an ultrasonic bath (Bransonic TM 200, Branson Ultrasonics Corporation, Danbury, CT, USA) and their temperature adjusted at 20 °C. Afterwards, 50 mL were placed in a glass chromatography column (25 mm diameter, 500 mm in length, with a 16–40 μm glass, sintered disc at the base) equipped with a 2-mm bore glass valve. A plastic pipe connected the column with a flow regulator set at 100 mL/min and this to a CO_2_ cylinder set at 1 bar pressure. After pouring, the wine height was recorded as the starting point. After the CO_2_ flow started, the height of the produced foam was recorded at thirty-second intervals for 10 min to calculate the maximum foam height (HM) and the foam stability height (HS), as well as the time taken for the foam at the center of the wine surface to dissipate once the CO_2_ flow had been terminated (TS). Three bottles for each wine sample were analyzed in duplicate.

### 2.9. Viscosity Analysis

The rheological analysis was performed using a rotational rheometer (Kinexus Pro+, Malvern, UK) at a constant temperature of 16 °C (±0.03 °C). The conical geometry was 60 mm in diameter with a cone angle of 1°. Experimental procedure and validation of the protocol were detailed by Halefadl et al. [28]. Once the rheometer was calibrated for 20 min in air, the samples were loaded onto the plate, ensuring no bubbles were entrapped in the sample and that any excess sample was removed. The revolution of the geometry was set to 100 rpm (shear rate 93 s^−1^) for 20 min per analysis using rSpace software (version 1.3). Each sample was tested in triplicate, and the viscosity was measured as the dynamic viscosity (mPa/s).

### 2.10. Sensory Assessment

Tasting took place 12 months after tirage at the Rathfinny Research Winery (Plumpton College). The panel, consisting of 7 judges aged between 20 and 50, all with extensive wine descriptive analysis experience, was asked to evaluate the intensity of the perceived attributes by means of an internal descriptive protocol [29] that established a different range scale for each attribute. The attributes were as follows: Appearance and Color (range scale: 0–2), Effervescence (0–3), Aroma and Bouquet (0–4), Acidity (0–1), Balance (0–2), Flavor (0–3), Finish (0–2) and Overall Quality (0–3). A clear and extensive description of each score for each attribute was available to the judges. Finally, the judges were requested to rank the wines according to their pleasantness. Four (Wines B and C) and five (Wines A) wines were evaluated during each session. For both the descriptive analysis and the preference test, the wines were presented according to a randomized complete block design. Samples (30 mL) coded with 3-digit numbers were presented at 6 °C in 170-mL tulip glasses [30] covered with transparent plastic dishes to preserve the aroma. Panelists were allowed to rinse their mouth by drinking water and eating unsalted crackers between samples.

### 2.11. Statistical Analyses

The chemical data were processed, statistically analyzed and visualized using GraphPad Prism software version 7.05 (GraphPad Software, San Diego, CA, USA). One-way analysis of variance (ANOVA) followed by a post hoc Tukey test was used to determine the statistical significance using a *p*-value of 0.05. A Principal Component Analysis (PCA) of the phenolics was performed using XLSTAT version 2011.1.05 (Addinsoft, Anglesey, UK) at a significant *p*-value ≤ 0.05. As for the sensory analyses, the data were first analyzed to disclose differences in pleasantness among the wines by means of the Kruskal–Wallis rank sum; then, an ordinal logistic regression model was used for the multivariate analysis to identify those sensory descriptors that affected the pleasantness in the wines. All analyses were performed at a significant *p*-value ≤ 0.05 using XLSTAT version 2011.1.05 (Addinsoft, Anglesey, UK).

## 3. Results and Discussion

Depending on the strain of yeast used to carry out the SiBAF, the compositions of the finished wines can vary, as different yeast strains can lead to differences in fermentation kinetics, in the release of non-volatile and volatile compounds and in autolytic characteristics [3,31,32]. Therefore, the data on the most important parameters related to sparkling wine quality, namely wines’ chemical and macromolecular compositions, phenolic profiles, foaming and viscosity properties, were determined after a 1-year period of aging on lees from 13 wines produced starting from three base wines and by using a total of five yeast strains (see Figure 1).

### 3.1. Yeast Strain Effect on Wine Chemical Composition

The compositional parameters of the 13 wines studied are reported in Table 2.

In general, the use of different strains on the three base wines did not cause noticeable modifications on the main wine chemical parameters, as the alcohol strength (ethanol), pH, titratable acidity, organic acid profile and SO_2_ (free and total) did not vary significantly. While data on the fermentation rates have not been recorded, the lack of differences in the ethanol contents indicates that the sugar/ethanol conversion rate of the interspecific yeast hybrids AWRI 1571, AWRI 1572, AWRI 2526 and AWRI 1502 does not differ from that of the *S. cerevisiae* strains AWRI 1616 and IOC 18-2007. However, the residual sugar content for each of the three wines showed some small variations, as the values of glucose and, especially, fructose were always slightly but significantly higher than those found in wines fermented with commercial yeast S. cerevisiae strains (IOC 18-2007and AWRI 1616). However, these residual sugars contents were still very low, as their sum never exceeded 2 g/L, a value that is typical of dry wines.

Interestingly, the acidity of the wines and the organic acid content were unaffected by the strain used. While different yeast strains are known to be able to modify wine acidity by differential release or the consumption of organic acids in wine [33], the lack of differences noted indicates that, at least in our experimental conditions, the impact on these parameters can be considered negligible.

### 3.2. Yeast Strain Effect on Wine Color and Phenolic Profiles

Differences in the wines’ phenolic contents and profiles were mostly attributable to the type and quantity of phenolics present in the grapes used to produce them and to the extent of their extraction during winemaking. However, yeast strains can also affect wines’ phenolic profiles by using different mechanisms. Indeed, during fermentation yeasts can release enzymes that can hydrolyze phenolic glycosides and can also adsorb phenolic compounds into their cell walls. Moreover, during autolysis, yeasts release compounds that are further able to modify the wine phenolic composition, such as mannoproteins that can adsorb phenolics, glutathione that prevents phenolic oxidation, β-glucosidase, which can modify wine phenolics [3,34,35]. Therefore, the wines were submitted to the CIELAB and HPLC analyses to gather information on the potential impacts of the yeast strains on the color (Table 3) and phenolic profiles (Table 4).

Table 3 shows the calculated ΔE*_ab_ values, which are the measures of differences in colors between samples calculated for pairs of the treatments. Pairs with values of ΔE*_ab_ greater than 1 are likely to be detected as different by the human eye [15]. None of the ΔE*_ab_ were greater than 0.5, indicating that the potential color differences associated with the different yeast strains used were invisible to the human eye. Therefore, the yeast strains used did not significantly alter the wine colors, a finding particularly relevant for wine C, as it was a rosé.

**Figure 2:**
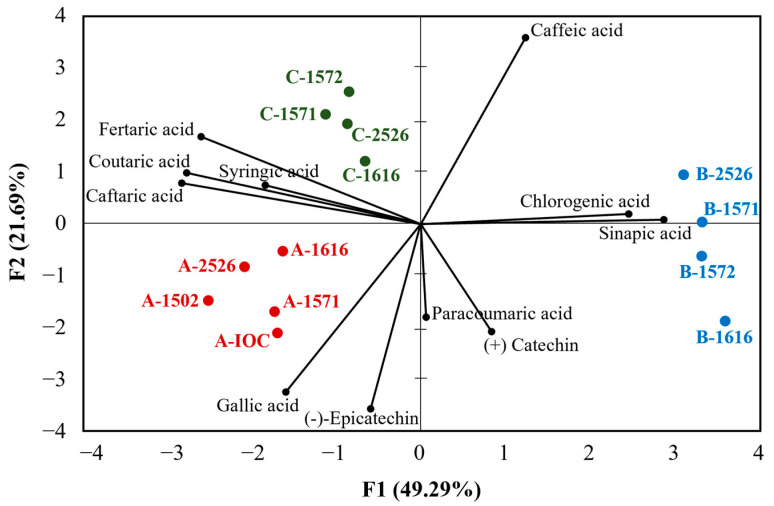
PCA Biplot of the sparkling wines (scores) obtained from SiBAF conducted using commercial *S. cerevisiae* or interspecific yeast hybrids and phenolic compounds (loading).

Despite the potential differences that yeast strains can induce with respect to the phenolic composition of a wine, the results reported in Table 4 show that the yeasts did not significantly impact the parameters considered, as the brown pigments, total phenolics, hydroxycinnamates and flavonoids were unaffected by the strain used. When considering the grape varieties used for the preparation of the three base wines, some considerations can be made. Wine A, the one with the highest values for all parameters considered in Table 4, was prepared using 59% of base wines from red varieties naturally richer in phenolic compounds than white varieties. This, together with vinification steps that could have extracted more phenolics from the grape solids (e.g., pressing) [3], could justify the higher values observed for wine A when compared to wine B, which, on the other hand, was made predominantly with Chardonnay. Wine C was produced exclusively from red grapes, and so, its intermediate total phenolic content was likely due to milder processing methods (e.g., lower pressures to extract the juice) than those applied for wine A. The phenolic profiles of the 13 wines were further investigated to ascertain the potential effects of the yeasts on the phenolic acids and simple phenols (gallic, syringic fertaric, paracoumaric, sinapic, caffeic, chlorogenic acids, (−) epicatechin and (+) catechin) compositions. The results were represented by means of a PCA, which explained 71% of the variability with the first two components (F1 = 49.29%; F2 = 21.69%) (Figure 2).

The differences in the phenolic compositions of the base wines allowed them to be clearly grouped together on different quadrants of the PCA. Indeed, wines A (lower/left side of the biplot) showed the highest values for the majority of the phenolics, namely gallic (range 27.7–33.0 mg/L), coutaric (1.1–1.3 mg/L), syringic (2.9–4.5 mg/L) and caftaric (13.0–16.2 mg/L) acids and (−) epicatechin (15.8–17.7 mg/L). All wines belonging to group B (right side of the biplot) were characterized by a higher quantity of chlorogenic (range 4.1–4.8 mg/L) and sinapic (6.0–6.6 mg/L) acids, while the wines from C (upper/left side) were distinguished thanks to their fertaric (0.7–9.8 mg/L) and caffeic (1.0–1.3 mg/L) contents. Therefore, while the strain of yeast could potentially affect the phenolic profile of a wine [3,34,35], the PCA clearly revealed that the “base wine” effect was predominant, and therefore, the potential impact of the yeast strains on the phenolic compositions of the wines could be considered negligible, a conclusion in agreement with the results reported in Table 3 and Table 4.

### 3.3. Yeast Strain Effect on Wine Macromolecular Composition and Wine Viscosity

Table 5 shows the parameters related to the macromolecular compositions of the 13 wines.

The three base wines showed different protein concentrations, with wine A reaching the highest value (range 60–91 mg/L), followed by wine C (range 15–22 mg/L). Almost no proteins were found in wine B (range 0.4–5.15 mg/L). Likely, these results were greatly affected by the different protein stabilization strategies of the base wines. Indeed, it can be assumed that base wine B would have been fully protein-stabilized, while wine C and especially A did not undergo such stabilization, or, if they did, it was only partial. Indeed, it is well known that the compounds released by the yeasts during aging on lees are responsible for improved protein stability of the wines [32], and therefore, bases for sparkling wines tend to be not fully stabilized. Additionally, the protein results indicate that the yeasts had a role in modulating their contents. Indeed, when wine A was refermented with yeast strain AWRI 1502, its protein content was significantly higher than the other four wines. The yeast effect was also visible in wine C, for which the wine fermented with AWRI 1571 showed a significantly lower protein content than the other three wines. Wine B contained very little proteins, but some significant differences were, however, noticeable, with only the wine fermented with AWRI 1572 showing a protein content significantly higher than the commercial control AWRI 1616. Interestingly, the lowest protein content for the three wines was always in those fermented with AWRI 1571, indicating that this yeast potentially secretes less proteins than the other strains used.

**Figure 3:**
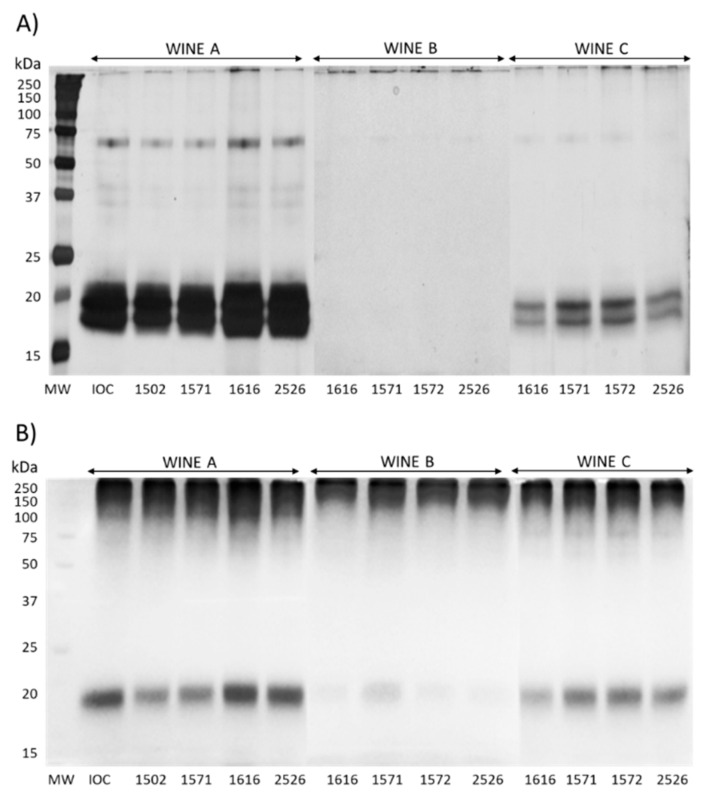
Sodium dodecyl sulfate-polyacrylamide gel electrophoresis analysis on the reducing conditions of the 13 wines. (**A**) Silver-stained gels to visualize the proteins, and (**B**) gels stained with the periodic acid–Schiff (PAS) method for the visualization of sugar-containing molecules. MW, molecular weight standard proteins.

When looking at the polysaccharide content (Table 5), the three wines showed total values in line with those reported in the literature [36,37]. For this parameter, the role of the yeast strain was less important than that observed for the proteins. Indeed, the yeast strains did not induce many major modifications, except for wine B wines fermented with AWRI 2526 and AWRI 1572, which contain about 13% more polysaccharides than the control AWRI 1616. Wine A was the one with the highest content of polysaccharides, while wines B and C contained similar amounts. As discussed in the commentary of Table 4, in this case, the contents of the polysaccharides also seem to be mostly attributable to differences in the base wines, an occurrence that can be due to a variety of factors, including the ripeness level of the grapes and the extraction of these compounds from grape solids due to the processing methods used (e.g., pressing) [38]. Nevertheless, some variations linked to the strain used were visible in wine B, suggesting that, at least under certain conditions, the interspecific hybrids can release more polysaccharides in the wine than the *S. cerevisiae* strains. It is well known that most polysaccharides found in wine are mannoproteins (MPs) [39] and even more so in sparkling wines, as they are believed to be enriched with these compounds by the long autolytic period during lees aging [32]. This was confirmed by the results presented here on the total MP contents for wine A, as these indicate that different yeast strains led to significant differences for this parameter that ranged between 123.8 mg/L and 303.2 mg/L. Despite not being measured, MPs were certainly also present in wines B and C, as visible from the data and the discussion related to Figure 3. Given that MPs impact several wine qualities such as stability, color, the foaming properties and aroma modulation [40,41,42,43], it is noteworthy to notice how these strains led to dramatic modifications for this parameter.

In terms of wine viscosity, a parameter that could potentially be affected by the compounds that yeasts release in wine during aging (e.g., polysaccharides and glycerol), the reported values were in line with those available in the literature [13,27,44,45], while no significant differences were found in any of the three wines tested. This finding indicates that the contribution of the strains used for SiBAF was too small to result in significant modifications of this parameter. To better understand the potential role of macromolecules on wine quality, the 13 wine samples were analyzed by electrophoresis (Figure 3).

The intensity of the bands in Figure 3A is in line with the protein concentration data (see Table 5). In general, the protein profiles of these wines are typical of those of white wines [46] and include a protein band at around 65 kDa (likely invertases) and two major bands at around 20 kDa, tentatively identified as chitinases and thaumatin-like proteins.

When comparing the intensity of the bands in Figure 3B with the polysaccharides and MP concentrations shown in Table 5, a correspondence is noticeable, with wine A showing the darkest bands, followed by wines C and B. It can be noticed that the wines had similar glycosylated compounds profiles, with the visible bands possessing different mobilities and intensities. When analyzing the glycoproteins by electrophoresis, visible bands are necessarily charged proteins; otherwise, they would not migrate in the gel. Therefore, the bands in Figure 3B can be considered MPs, as these are the sole charged glycoproteins in the wine able to migrate in the gel [18]. When looking at the intensity of the bands for wine A, it seems that this is in line with the MPs quantification data shown in Table 5, with AWRI1616 showing the highest intensity and AWRI1502 and AWRI1571 the lowest. While the MPs concentration data for wines B and C are not available, the bands’ intensities in Figure 3B suggest that wine C contained less MPs than wine A but more than wine B. Additionally, in this case, differences in the band intensities linked to the yeast strain used were visible, confirming the impact on the MPs released by the yeast observed for wine A.

### 3.4. Yeast Strain Effect on Wine Foamability

**Figure 4:**
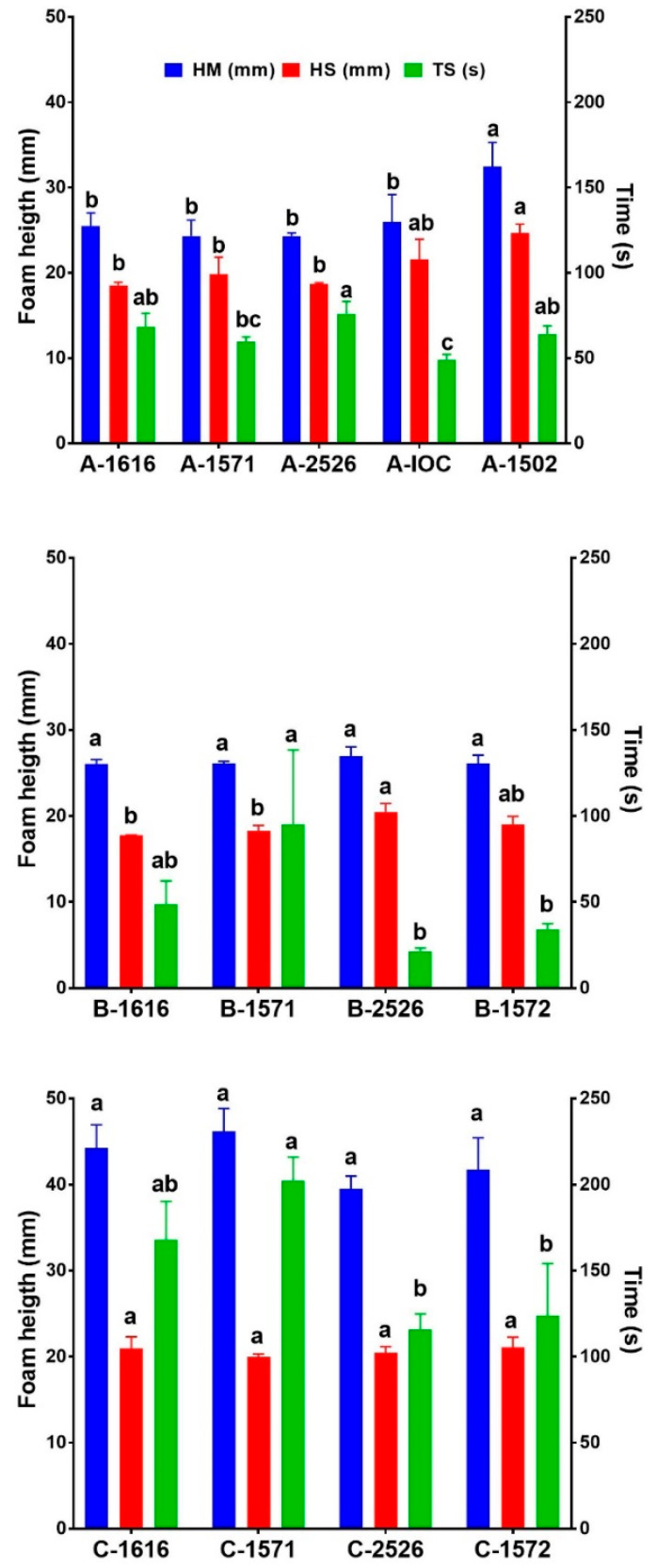
Effects of yeast type on the mean foam height (HM, blue bars), foam stability (HS, red bars) and time stability (TS, green bars). Error bars represent the SD. Different letters represent statistically significant differences between treatments (post hoc Tukey test, *p* = 0.05); n = 3 per treatment.

The foam properties of sparkling wines are known to be significantly affected by the wine chemical composition in general and by the wine macromolecules (e.g., proteins and polysaccharides) in particular [47]. Therefore, the yeasts’ impacts on key foam attributes were assessed by using the modified Mosalux method (Figure 4).

The two white wines (A and B) had very similar mean foam heights (HM) (26.5 mm vs. 26.3 mm), while the rosé wine (wine C) had an average HM that was 42.9 mm, a finding in line with the data from Martínez-Lapuente et al., who reported a positive correlation between HM and anthocyanins in rosé wines [48]. Within each set of wines, no significant impact on HM by the yeast was visible, with the exception of yeast AWRI 1502 in wine A, for which the HM was significantly higher than the other four wines (F(4,10) = 7.124, *p* = 0.0056), a result that could be explained by the higher protein content of wine A-1502 (see Table 5), in agreement with the literature reports [49]. These data indicate that the base wine used had a bigger impact on the average HM than the yeast used for SiBAF.

The foam stability (HS) values were very similar among the three wines. For HS, the yeast strain played a role on only two occasions: wine A refermented with the yeast AWRI 1502 and wine B refermented with yeast AWRI 2526 both possessed a significantly stabler foam (F(4,10) = 8.88, *p* = 0.0025 and F(4,10) = 34.93, *p* < 0.0001, respectively). For wine A, this finding could be ascribed to its high protein content compared to the other wines, while, for wine B, this increased stability could be due to the higher content of polysaccharides in this wine (Table 5), as this class of compounds has been reported to increase the foam stability [50,51].

The third parameter measured, time stability (TS), showed a more heterogeneous behavior among the wines, with wine C showing the highest average TS (152.5 s), followed by wine A (63.4 s) and wine B (38.7 s). By looking at the TS for each of the three wines individually, it could be noticed that, for wine A, the only two strains that induced a significantly shorter TS were AWRI 1571 and IOC-18-2007 when compared to AWRI1616 taken as the reference for this experiment. For wines B and C, every strain used was not significantly different from AWRI1616.

### 3.5. Yeast Strain Effect on Wine Sensory Profile

Yeasts are known to release volatile aroma compounds into wine during both fermentation and autolysis [3]. Therefore, the potential impact of the strain used for SiBAF on the sensory profiles of the thirteen wines under investigation was assessed.

In wine A, the ranking test showed that the wines from SiBAF carried out with commercial IOC-18-2007 and AWRI 1502 obtained the highest pleasantness when compared to wines produced with a commercial control strain (AWRI 1616) or interspecific yeast hybrids (AWRI 1571 and AWRI 2526). Interestingly, these three wines showed similar pleasantness values. Ordinal logistic regression identified “aroma and bouquet” (*p* = 0.018), “balance” (*p* = 0.05), “finish” (*p* = 0.006) and “overall quality” (*p* = 0.016) as the sensory descriptors significantly different and therefore possibly being responsible for the higher ranking of the IOC-18-2007 and AWRI 1502 wines.

In wine B, the commercial IOC-18-2007 and AWRI 1502 strains, which produced the best ratings for wine A, were not considered, and in this case, the commercial yeast AWRI 1616 and the interspecific yeast hybrid AWRI 1571 showed higher preferences compared to the wines from commercial AWRI 1572 and interspecific yeast hybrid AWRI 2526. The sensory descriptor responsible for the difference between the two groups of wines was “acidity” (*p* = 0.005), which was higher in the AWRI 1616 and interspecific yeast hybrid AWRI 1571 wines.

In wine C, where the same yeasts as in wine B were used, no differences in terms of pleasantness were detected among the wines.

These results indicate that the sensory characteristics and general pleasantness of wines are affected by the choice of the yeast strain, modifying different attributes such as “aroma and bouquet”, “finish”, “balance” and “overall quality”. In our study, the wines were mostly different when using the SiBAF IOC-18-2007 and AWRI 1502, whereas the differences between strains AWRI 1616, 1572, 1571 and 2526 could only be noted in one set of wines. In none of the tested wines could a difference between AWRI 1616 and AWRI 1571 be detected.

## 4. Conclusions

The attempt to produce, starting from commercial base wines, bottle-fermented sparkling wines with alternative yeast strains was overall satisfactory and indicated that interspecific yeast hybrids can be used for the elaboration of sparkling wines, as they provided wines with chemical and sensory characteristics generally similar to those produced with commonly used commercial *Saccharomyces cerevisiae* strains. Indeed, the findings indicate that the novel interspecific yeast hybrids can successfully conduct a SiBAF to produce dry wines without substantial modifications in terms of the wine chemical characteristics when compared to the commonly used commercial *Saccharomyces cerevisiae* strains. The choice of yeast led to some changes in the parameters related to the autolytic properties of different strains, with different releases in proteins and polysaccharides that varied in the total amounts, as well as in composition, with some strains releasing more mannoproteins into the wine than others, an occurrence likely to influence several important wine quality attributes such as color and foamability, as well as the volatile profile and sensory perception. Therefore, future studies will need to consider additional parameters such as the volatile composition, foaming properties and sensorial aspects of wines produced from different varieties and by potentially also using other production methods such as tank fermentation.

## Figures and Tables

**Table 1:** Overview of the commercial yeast and interspecific hybrids used for the secondary in-bottle alcoholic fermentation.

Commercial Name	Yeast Strain	Producer
IOC 18-2007	*Saccharomyces cerevisiae*	Institut Œnologique de Champagne, Mardeuil, France
AWRI 1616 (PDM)	*Saccharomyces cerevisiae*	AB Mauri Yeast, Camellia, Australia
AWRI 2526	*Saccharomyces cerevisiae x mikatae*	AB Mauri Yeast, Camellia, Australia
AWRI 1572	*Saccharomyces cerevisiae x bayanus*	AB Mauri Yeast, Camellia, Australia
AWRI 1571	*Saccharomyces cerevisiae x bayanus*	AB Mauri Yeast, Camellia, Australia
AWRI 1502	*Saccharomyces cerevisiae x cariocanus*	AB Mauri Yeast, Camellia, Australia

**Table 2:** Wine composition parameters. Mean values are shown (n ≥ 3). For each wine and within each row, means followed by a different letter are significantly different (*p* = 0.05), according to the post hoc Tukey test.

	Wine A—Blanc de Blancs					Wine B—Blanc de Blancs				Wine C—Rosé			
	AWRI 1616	AWRI 1571	AWRI 2526	AWRI 1502	IOC 18- 2007	AWRI 1616	AWRI 1571	AWRI 2526	AWRI 1572	AWRI 1616	AWRI 1571	AWRI 2526	AWRI 1572
Ethanol (% vol)	12.80 ^a^	12.60 ^a^	12.67 ^a^	12.47 ^a^	12.73 ^a^	12.87 ^a^	12.83 ^a^	12.87 ^a^	12.83 ^a^	13.40 ^a^	13.23 ^a^	13.33 ^a^	13.37 ^a^
Glucose (g/L)	0.10 ^d^	0.13 ^c^	0.16 ^b^	0.20 ^a^	0.11 ^d^	0.11 ^c^	0.15 ^ab^	0.18 ^a^	0.13 ^bc^	0.14 ^c^	0.21 ^ab^	0.27 ^a^	0.18 ^bc^
Fructose (g/L)	0.35 ^e^	0.71 ^c^	0.80 ^b^	1.37 ^a^	0.48 ^d^	0.36 ^c^	0.57 ^bc^	0.95 ^a^	0.65 ^b^	0.48 ^c^	1.21 ^b^	1.7 ^a^	1.16 ^b^
SO_2_ (free) (mg/L)	29 ^a^	31 ^a^	29 ^a^	28 ^a^	29 ^a^	15 ^a^	15 ^a^	18 ^a^	16 ^a^	17 ^a^	18 ^a^	18 ^a^	16 ^a^
SO_2_ (total) (mg/L)	118 ^a^	116 ^a^	112 ^a^	114 ^a^	133 ^a^	69 ^a^	70 ^a^	70 ^a^	70 ^a^	83 ^a^	83 ^a^	96 ^a^	86 ^a^
pH	3.22 a	3.23 ^a^	3.24 ^a^	3.21 ^a^	3.23 ^a^	3.28 ^a^	3.29 ^a^	3.28 ^a^	3.28 ^a^	3.14 ^a^	3.14 ^a^	3.14 ^a^	3.14 ^a^
Titratable acidity (g/L)	9.18 ^a^	8.70 ^a^	9.10 ^a^	8.90 ^a^	9.20 ^a^	8.11 ^a^	7.90 ^a^	7.87 ^a^	8.11 ^a^	9.00 ^a^	9.10 ^a^	9.00 ^a^	9.00 ^a^
Citric acid (g/L)	0.17 ^a^	0.17 ^a^	0.17 ^a^	0.18 ^a^	0.19 ^a^	0.04 ^a^	0.04 ^a^	0.04 ^a^	0.04 ^a^	0.13 ^a^	0.13 ^a^	0.12 ^a^	0.12 ^a^
Tartaric acid (g/L)	2.4 1^a^	2.37 ^a^	2.39 ^a^	2.40 ^a^	2.37 ^a^	1.82 ^a^	1.83 ^a^	1.84 ^a^	1.85 ^a^	1.80 ^a^	1.78 ^a^	1.80 ^a^	1.78 ^a^
Malic acid (g/L)	4.67^a^	4.67 ^a^	4.61 ^a^	4.65 ^a^	4.45 ^a^	2.28 ^a^	2.27 ^a^	2.29 ^a^	2.31 ^a^	4.87 ^a^	4.86 ^a^	4.88 ^a^	4.88 ^a^
Succinic acid (g/L)	1.69^a^	1.68 ^a^	1.71 ^a^	1.67 ^a^	1.66 ^a^	3.10 ^a^	3.14 ^a^	3.17 ^a^	3.19 ^a^	1.29 ^a^	1.29 ^a^	1.33 ^a^	1.30 ^a^
Lactic acid (g/L)	1.02 ^a^	0.99 ^a^	1.01 ^a^	1.04 ^a^	1.00 ^a^	3.82 ^a^	3.63 ^a^	3.72 ^a^	3.81 ^a^	1.41 ^a^	1.52 ^a^	1.40 ^a^	1.40 ^a^
Acetic acid (g/L)	0.19 ^a^	0.30 ^a^	0.23 ^a^	0.22 ^a^	0.20 ^a^	0.36 ^a^	0.26 ^a^	0.30 ^a^	0.36 ^a^	0.19 ^a^	0.16 ^a^	0.21 ^a^	0.22 ^a^

**Table 3:** Mean CIELAB ΔE*_ab_ values for wines. ΔE*_ab_ = √[(L1 − L2)^2^ + (a1 − a2)^2^ + (b1 − b2)^2^]. CIELAB, Commission Internationale de l’Eclairage Lab transmission values L*, a* and b*. ΔE*_ab_ < 1 means a normally invisible difference.

Wine A—Blanc de Blancs	ΔE*_ab_	Wine B—Blanc de Blancs	ΔE*_ab_	Wine C—Rosé	ΔE*_ab_
AWRI 1616 vs. AWRI 1571	0.4	AWRI 1616 vs. AWRI 1571	0.2	AWRI 1616 vs. AWRI 1571	0.5
AWRI 1616 vs. AWRI 2526	0.0	AWRI 1616 vs. AWRI 2526	0.2	AWRI 1616 vs. AWRI 2526	0.4
AWRI 1616 vs. AWRI 1502	0.2	AWRI 1616 vs. AWRI 1572	0.1	AWRI 1616 vs. AWRI 1572	0.4
AWRI 1616 vs. IOC 18-2007	0.1	AWRI 1571 vs. AWRI 2526	0.4	AWRI 1571 vs. AWRI 2526	0.3
AWRI 1571 vs. AWRI 2526	0.4	AWRI 1571 vs. AWRI 1572	0.2	AWRI 1571 vs. AWRI 1572	0.2
AWRI 1571 vs. AWRI 1502	0.5	AWRI 2526 vs. AWRI 1572	0.2	AWRI 2526 vs. AWRI 1572	0.2
AWRI 1571 vs. IOC 18-2007	0.3				
AWRI 2526 vs. AWRI 1502	0.2				
AWRI 2526 vs. IOC 18-2007	0.1				
AWRI 1502 vs. IOC 18-2007	0.3				

**Table 4:** Wine phenolic composition parameters. Mean values are shown (n ≥ 3). For each wine and within each row, means followed by a different letter are significantly different (*p* = 0.05), according to the post hoc Tukey test.

	Wine A—Blanc de Blancs					Wine B—Blanc de Blancs				Wine C—Rosé			
	AWRI 1616	AWRI 1571	AWRI 2526	AWRI 1502	IOC 18- 2007	AWRI 1616	AWRI 1571	AWRI 2526	AWRI 1572	AWRI 1616	AWRI 1571	AWRI 2526	AWRI 1572
Brown pigments	0.088 ^a^	0.125 ^a^	0.084 ^a^	0.099 ^a^	0.084 ^a^	0.044 ^a^	0.062 ^a^	0.051 ^a^	0.059 ^a^	0.073 ^a^	0.073 ^a^	0.084 ^a^	0.095 ^a^
Total phenolics (AU)	3.319 ^a^	2.538 ^a^	3.385 ^a^	3.168 ^a^	3.051 ^a^	1.236 ^a^	1.368 ^a^	1.082 ^a^	0.924 ^a^	1.581 ^a^	1.372 ^a^	1.045 ^a^	1.581 ^a^
Total Hydroxycinnamates (AU)	2.373 ^a^	2.168 ^a^	2.329 ^a^	2.384 ^a^	2.520 ^a^	1.141 ^a^	1.181 ^a^	1.108 ^a^	1.277 ^a^	1.878 ^a^	1.827 ^a^	1.786 ^a^	1.900 ^a^
Total flavonoids (AU)	1.752 ^a^	1.107 ^a^	1.848 ^a^	1.595 ^a^	1.388 ^a^	0.483 ^a^	0.588 ^a^	0.351 ^a^	0.082 ^a^	0.341 ^a^	0.166 ^a^	0.134 ^a^	0.327 ^a^

**Table 5:** Macromolecules and viscosity values. Mean values are shown (n ≥ 3). For each wine and within each row, means followed by a different letter are significantly different (*p* = 0.05), according to the post hoc Tukey test. n.m., not measured.

	Wine A—Blanc de Blancs					Wine B—Blanc de Blancs				Wine C—Rosé			
	AWRI 1616	AWRI 1571	AWRI 2526	AWRI 1502	IOC 18- 2007	AWRI 1616	AWRI 1571	AWRI 2526	AWRI 1572	AWRI 1616	AWRI 1571	AWRI 2526	AWRI 1572
Protein (mg/L)	63.1 ^a^	60.8 ^a^	63.7 ^a^	91.1 ^b^	69.3 ^a^	1.5 ^ab^	0.4 ^a^	2.9 ^b^	5.1 ^c^	20.9 ^b^	15.1 ^a^	21.9 ^b^	20.5 ^b^
Polysaccharides (mg/L)	352.4 ^a^	332.8 ^a^	327.2 ^a^	334.5 ^a^	361.3 ^a^	218.5 ^a^	227.9 ^ab^	246.4 ^b^	246.1 ^b^	243.7 ^a^	243.1 ^a^	263.9 ^a^	241.5 ^a^
Mannoproteins (mg/L)	303.2 ^a^	167.8 ^b^	158.9 ^b^	199.1 ^b^	123.8 ^d^	n.m.	n.m.	n.m.	n.m.	n.m.	n.m.	n.m.	n.m.
Viscosity (mPa/s)	1.91 ^a^	1.92 ^a^	1.98 ^a^	1.96 ^a^	2.02 ^a^	1.96 ^a^	1.98 ^a^	1.98 ^a^	1.96 ^a^	1.99 ^a^	1.98 ^a^	2.00 ^a^	1.97 ^a^

## Data Availability

The data presented in this study are available upon request from the corresponding author.

## References

[1] OIV (2022). International Code of Oenological Practices.

[2] OIV (2020). OIV Focus: The Global Sparking Wine Market.

[3] Kemp B., Alexandre H., Robillard B., Marchal R. (2015). Effect of Production Phase on Bottle-Fermented Sparkling Wine Quality. J. Agric. Food Chem..

[4] Ivit N.N., Kemp B. (2018). The Impact of Non-*Saccharomyces* Yeast on Traditional Method Sparkling Wine. Fermentation.

[5] Alexandre H., Romano P., Ciani M., Fleet G. (2019). Yeasts and Sparkling Wine Production. Yeasts in the Production of Wine.

[6] Tofalo R., Perpetuini G., Rossetti A.P., Gaggiotti S., Piva A., Olivastri L., Cichelli A., Compagnone D., Arfelli G. (2022). Impact of *Saccharomyces cerevisiae* and Non-*Saccharomyces* Yeasts to Improve Traditional Sparkling Wines Production. Food Microbiol..

[7] González-Royo E., Pascual O., Kontoudakis N., Esteruelas M., Esteve-Zarzoso B., Mas A., Canals J.M., Zamora F. (2015). Oenological Consequences of Sequential Inoculation with Non-*Saccharomyces* Yeasts (*Torulaspora Delbrueckii* or *Metschnikowia Pulcherrima*) and *Saccharomyces cerevisiae* in Base Wine for Sparkling Wine Production. Eur. Food Res. Technol..

[8] Canonico L., Comitini F., Ciani M. (2018). *Torulaspora Delbrueckii* for Secondary Fermentation in Sparkling Wine Production. Food Microbiol..

[9] Capozzi V., Tufariello M., Berbegal C., Fragasso M., De Simone N., Spano G., Russo P., Venerito P., Bozzo F., Grieco F. (2022). Microbial Resources and Sparkling Wine Differentiation: State of the Arts. Fermentation.

[10] Bellon J.R., Schmid F., Capone D.L., Dunn B.L., Chambers P.J. (2013). Introducing a New Breed of Wine Yeast: Interspecific Hybridisation between a Commercial *Saccharomyces cerevisiae* Wine Yeast and *Saccharomyces mikatae*. PLoS ONE.

[11] Bellon J.R., Ford C.M., Borneman A.R., Chambers P.J. (2018). A Novel Approach to Isolating Improved Industrial Interspecific Wine Yeasts Using Chromosomal Mutations as Potential Markers for Increased Fitness. Front. Microbiol..

[12] Borneman A.R., Forgan A.H., Kolouchova R., Fraser J.A., Schmidt S.A. (2016). Whole Genome Comparison Reveals High Levels of Inbreeding and Strain Redundancy Across the Spectrum of Commercial Wine Strains of *Saccharomyces cerevisiae*. G3.

[13] Crumpton M., Rice C.J., Atkinson A., Taylor G., Marangon M. (2018). The Effect of Sucrose Addition at Dosage Stage on the Foam Attributes of a Bottle-Fermented English Sparkling Wine. J. Sci. Food Agric..

[14] Iland P., Bruer N., Edwards G., Weeks S., Wilkes E. (2004). Chemical Analysis of Grapes and Wine: Techniques and Concepts.

[15] Kwiatkowski M.J., Skouroumounis G.K., Lattey A.A., Waters E.J. (2007). The Impact of Closures, Including Screw Cap with Three Different Headspace Volumes, on the Composition, Colour and Sensory Properties of a Cabernet Sauvignon Wine during Two Years’ Storage. Aust. J. Grape Wine Res..

[16] Vincenzi S., Mosconi S., Zoccatelli G., Pellegrina C.D., Veneri G., Chignola R., Peruffo A., Curioni A., Rizzi C. (2005). Development of a New Procedure for Protein Recovery and Quantification in Wine. Am. J. Enol. Vitic..

[17] Segarra I., Lao C., López-Tamames E., De La Torre-Boronat M.C. (1995). Spectrophotometric Methods for the Analysis of Polysaccharide Levels in Winemaking Products. Am. J. Enol. Vitic..

[18] Marassi V., Marangon M., Zattoni A., Vincenzi S., Versari A., Reschiglian P., Roda B., Curioni A. (2021). Characterization of Red Wine Native Colloids by Asymmetrical Flow Field-Flow Fractionation with Online Multidetection. Food Hydrocoll..

[19] Marangon M., Vegro M., Vincenzi S., Lomolino G., De Iseppi A., Curioni A. (2018). A Novel Method for the Quantification of White Wine Mannoproteins by a Competitive Indirect Enzyme-Linked Lectin Sorbent Assay (CI-ELLSA). Molecules.

[20] Mercurio M.D., Dambergs R.G., Herderich M.J., Smith P.A. (2007). High Throughput Analysis of Red Wine and Grape Phenolics—Adaptation and Validation of Methyl Cellulose Precipitable Tannin Assay and Modified Somers Color Assay to a Rapid 96 Well Plate Format. J. Agric. Food Chem..

[21] Ivanova-Petropulos V., Ricci A., Nedelkovski D., Dimovska V., Parpinello G.P., Versari A. (2015). Targeted Analysis of Bioactive Phenolic Compounds and Antioxidant Activity of Macedonian Red Wines. Food Chem..

[22] Marangon M., Lucchetta M., Waters E.J. (2011). Protein Stabilisation of White Wines Using Zirconium Dioxide Enclosed in a Metallic Cage. Aust. J. Grape Wine Res..

[23] Laemmli U.K. (1970). Cleavage of Structural Proteins during the Assembly of the Head of Bacteriophage T4. Nature.

[24] Blum H., Beier H., Gross H.J. (1987). Improved Silver Staining of Plant Proteins, RNA and DNA in Polyacrylamide Gels. Electrophoresis.

[25] Doerner K.C., White B.A. (1990). Detection of Glycoproteins Separated by Nondenaturing Polyacrylamide Gel Electrophoresis Using the Periodic Acid-Schiff Stain. Anal. Biochem..

[26] Brissonet F., Maujean A. (1993). Characterization of Foaming Proteins in a Champagne Base Wine. Am. J. Enol. Vitic..

[27] Crumpton M., Atkinson A., Marangon M. (2018). Effect of Carboxymethyl Cellulose Added at the Dosage Stage on the Foamability of a Bottle-Fermented Sparkling Wine. Beverages.

[28] Halelfadl S., Estellé P., Aladag B., Doner N., Maré T. (2013). Viscosity of Carbon Nanotubes Water-Based Nanofluids: Influence of Concentration and Temperature. Int. J. Therm. Sci..

[29] Lawless H.T., Heymann H. (1998). Sensory Evaluation of Food: Principles and Practices.

[30] (1977). Sensory Analysi Apparatus: Wine-Tasting Glass.

[31] Sawyer S., Longo R., Solomon M., Nicolotti L., Westmore H., Merry A., Gnoinski G., Ylia A., Dambergs R., Kerslake F. (2022). Autolysis and the Duration of Ageing on Lees Independently Influence the Aroma Composition of Traditional Method Sparkling Wine. Aust. J. Grape Wine Res..

[32] Alexandre H., Guilloux-Benatier M. (2006). Yeast Autolysis in Sparkling Wine—A Review. Aust. J. Grape Wine Res..

[33] Vilela A. (2019). Use of Nonconventional Yeasts for Modulating Wine Acidity. Fermentation.

[34] Zhang P., Ma W., Meng Y., Zhang Y., Jin G., Fang Z. (2021). Wine Phenolic Profile Altered by Yeast: Mechanisms and Influences. Compr. Rev. Food Sci. Food Saf..

[35] Mekoue Nguela J., Poncet-Legrand C., Sieczkowski N., Vernhet A. (2016). Interactions of Grape Tannins and Wine Polyphenols with a Yeast Protein Extract, Mannoproteins and β-Glucan. Food Chem..

[36] Martínez-Lapuente L., Guadalupe Z., Ayestarán B., Ortega-Heras M., Pérez-Magariño S. (2013). Changes in Polysaccharide Composition during Sparkling Wine Making and Aging. J. Agric. Food Chem..

[37] Pons-Mercadé P., Giménez P., Vilomara G., Conde M., Cantos A., Rozès N., Ferrer S., Canals J.M., Zamora F. (2022). Monitoring Yeast Autolysis in Sparkling Wines from Nine Consecutive Vintages Produced by the Traditional Method. Aust. J. Grape Wine Res..

[38] Jones-Moore H.R., Jelley R.E., Marangon M., Fedrizzi B. (2021). The Polysaccharides of Winemaking: From Grape to Wine. Trends Food Sci. Technol..

[39] Waterhouse A.L., Sacks G.L., Jeffery D.W. (2016). Understanding Wine Chemistry.

[40] Pérez-Magariño S., Martínez-Lapuente L., Bueno-Herrera M., Ortega-Heras M., Guadalupe Z., Ayestarán B. (2015). Use of Commercial Dry Yeast Products Rich in Mannoproteins for White and Rosé Sparkling Wine Elaboration. J. Agric. Food Chem..

[41] Junior W.J.F.L., Nadai C., Rolle L., da Silva Gulão E., da Rocha Leão M.H.M., Giacomini A., Corich V., Vincenzi S. (2020). Influence of the Mannoproteins of Different Strains of *Starmenella bacillaris* Used in Single and Sequential Fermentations on Foamability, Tartaric and Protein Stabilities of Wines. Oeno One.

[42] Juega M., Nunez Y.P., Carrascosa A.V., Martinez-Rodriguez A.J. (2012). Influence of Yeast Mannoproteins in the Aroma Improvement of White Wines. J. Food Sci..

[43] Caridi A. (2006). Enological Functions of Parietal Yeast Mannoproteins. Antonie Van Leeuwenhoek.

[44] Neto F.S., de Castilhos M.B., Telis V.R., Telis-Romero J. (2015). Effect of Ethanol, Dry Extract and Reducing Sugars on Density and Viscosity of Brazilian Red Wines. J. Sci. Food Agric..

[45] Yanniotis S., Kotseridis G., Orfanidou A., Petraki A. (2007). Effect of Ethanol, Dry Extract and Glycerol on the Viscosity of Wine. J. Food Eng..

[46] Van Sluyter S.C., McRae J.M., Falconer R.J., Smith P.A., Bacic A., Waters E.J., Marangon M. (2015). Wine Protein Haze: Mechanisms of Formation and Advances in Prevention. J. Agric. Food Chem..

[47] Kemp B., Condé B., Jégou S., Howell K., Vasserot Y., Marchal R. (2019). Chemical Compounds and Mechanisms Involved in the Formation and Stabilization of Foam in Sparkling Wines. Crit. Rev. Food Sci. Nutr..

[48] Martínez-Lapuente L., Guadalupe Z., Ayestarán B., Pérez-Magariño S. (2015). Role of Major Wine Constituents in the Foam Properties of White and Rosé Sparkling Wines. Food Chem..

[49] Martínez-Lapuente L., Ayestarán B., Guadalupe Z., Jordão A.M., Cosme F. (2018). Influence of Wine Chemical Compounds on the Foaming Properties of Sparkling Wines. Grapes and Wines—Advances in Production, Processing, Analysis and Valorization.

[50] Andres-Lacueva C., Gallart M., Lopez-Tamames E., Lamuela-Raventos R.M., Andrés-Lacueva C. (1996). Influence of Variety and Aging on Foaming Properties of Sparkling Wine (Cava). J. Agric. Food Chem..

[51] Moreno-Arribas V., Pueyo E., Nieto F.J., Martín-Álvarez P.J., Polo M.C. (2000). Influence of the Polysaccharides and the Nitrogen Compounds on Foaming Properties of Sparkling Wines. Food Chem..

